# Assessment of Self-Perception of Transsexual Persons: Pilot Study of 15 Patients

**DOI:** 10.1155/2014/281326

**Published:** 2014-04-14

**Authors:** Jasmina Barišić, Marija Milosavljević, Dragana Duišin, Borjanka Batinić, Svetlana Vujović, Srdjan Milovanović

**Affiliations:** ^1^Clinic for Psychiatry, Clinical Centre of Serbia, 11000 Belgrade, Serbia; ^2^Department of Psychology, Faculty of Philosophy, University of Belgrade, 11000 Belgrade, Serbia; ^3^Clinic for Endocrinology, Clinical Centre of Serbia, 11000 Belgrade, Serbia; ^4^School of Medicine, University of Belgrade, 11000 Belgrade, Serbia

## Abstract

*Background and Aims*. There have been few studies in the area of Self-Perception in transsexual persons, except for the population of transsexual adolescents. Bearing in mind its importance not only in the assessment of personality but also in predicting adaptive capacity, the goal of our research is based on the examination of Self-Perception of adult transsexual persons. *Method*. The study was conducted using a Rorschach test, which provides an insight into various aspects of Self-Perception. The sample consisted of 15 transsexual persons, who passed the standard diagnostic procedure. *Results*. The results suggest that transsexual persons manage to maintain Adequate Self-Esteem. Hypervigilance Index and Obsessive Style Index are negative, while the values showing a negative quality of Self-Regard and the capacity for introspection tend to increase. In the process of Self-Introspection, negative and painful emotional states are often perceived. *Conclusion*. The estimation of Self-Perception in adult transsexual persons indicates a trend of subjective perception of a personal imperfection or inadequacy. This is probably the result of experiencing discomfort for a number of years due to gender incongruence and dysphoria, in particular in persons who enter the sex reassignment procedure later in their adulthood.

## 1. Introduction

According to the Tenth Revision of the International Classification of Diseases and Related Health Problems (ICD-10) [1] transsexualism (TS) has been an important research field for the last 30 years, given the specificity of persons with gender dysphoria (GD). Transsexual persons (TSP) are characterized by a strong and persistent sense of discomfort and inappropriateness with one's anatomic sex, identification with the opposite sex, and the persistent wish to be rid of one's genitals and live as a member of the other sex. Transsexualism is rare, with an estimated worldwide lifetime prevalence of 0.001-0.002% or 0.0019–0.0024% [2].

Previous research of transsexualism has been mainly focused on the assessment of psychiatric comorbidity with an emphasis on high risks for suicidal behavior and self-mutilation [3–5]. In addition, TS was associated with the presence of significant degree of personality psychopathology and severe psychiatric comorbidity, which has not been confirmed in recent studies [3, 4, 6–8].

There is predominance of the assessment scales and personality inventory in TS research studies with the most commonly used Minnesota Multiphasic Personality Inventory (MMPI-2), Mini-International Neuropsychiatric Interview (MINI), Structured Clinical Interview for DSM-IV Axis I and Axis II Disorders (SCID-I and SCID-II), and so forth [9–15].

Rorschach inkblot test has been rarely used in studies on TSP due to its specificity, complexity, and difficulties in interpretation, although the opportunities it provides are of major importance for the clinical assessment, in particular with the appearance of Exner's Comprehensive System [16]. The use of Exner's Comprehensive System provides an overview of the seven clusters involving major domains of psychological functioning (Processing, Mediation, Ideation, Controls, Affect, Self-Perception, and Interpersonal Perception) [16].

Review of the literature and research related to the assessment of Self-Perception of transseuxual persons is relatively modest. In one of the few studies dealing with this issue, Cohen et al. [17] find no support for the idea that adolescent transsexuals significantly differ from nonpatients with regard to thinking disturbances and negative Self-Image (score operationalized with the increased number of MOR responses). In another study which assessed pre- and postoperative functioning of adolescents no increase of Morbid (MOR) responses was observed, nor were other significant increases within the Self-Perception cluster [16]. These researches were focused on the evaluation of transsexual adolescents, whereas the authors of this study had no access to research concerning Self-Perception assessment of the adult transsexual persons.

Self-Perception is associated with Self-Image and Self-Involvement. Self-Image is made up of the impressions that the client has about his/her own characteristics. Many of these impressions are easily accessible to conscious thinking, but some are partially or completely out of reach of consciousness because they are unwanted or conflicting, so there is a tendency towards concealment or suppression.

Assessment of Self-Perception in Rorschach includes the assessment of the cluster Self-Esteem, Self-Regard, Self-Awareness, and Sense of Identity, and the resulting scores are interpreted by Exner's Comprehensive System [16, 18]. Global Self-Esteem is defined as an individual's personal Self-Esteem and how one feels about him or herself overall, that is, the individual's positive or negative attitude toward the self as totality [19]. Feeling satisfied with the characteristics people recognize in themselves gives them a sense of well-being and brings pleasure into their lives. Limited or impaired capacities to view themselves favorably and thoroughly make people susceptible to adjustment difficulties [18].

Transsexual persons are characterized by anatomic discomfort and accordingly a certain degree of subjective suffering due to gender, incongruence and dysphoria, while their stigmatization and minority stress (additive to general stressors experienced by all people) further emphasize the development of psychological symptoms, such as anxiety and depression [20, 21]. Lower global Self-Esteem has been also found as an effect of being a member of a devalued social group [22]. Bockting [23] notes that some negative outcomes for transsexual individuals may be due to the impact of social stigma and intrapsychical conflict between the individual's sex assigned at birth and gender identity. Therefore, it is possible to expect that Self-Perception in TSP often has a negative connotation, in particular in people who enter the sex reassignment procedure later in their adulthood.

In the assessment of Self-Perception of transsexual persons, besides the presence or absence of personality psychopathology, psychiatric comorbidity seems to be an important prognostic factor for long-term psychosocial adjustment [2].

Having in mind that studies in the field of Self-Perception with TSP are rare (except for the transsexual adolescents) and important in the process of personality assessment and prediction of adaptive capacity and outcomes during the transition and posttransition period, the goal of our research is based on the exploration of Self-Perception of adult transsexual persons. Even though there are a number of scales for Self-Perception assessment, we have decided to assess this concept by Rorschach method which provides an insight into various aspects of Self-Perception [19, 24].

## 2. Methods

### 2.1. Subjects

Fifteen patients who had been assessed and diagnosed with transsexualism according to the ICD-10 [1] at the Clinic for Psychiatry, Clinical Centre of Serbia, during one year, were requested to participate in this study, with the use of the Rorschach test. All patients underwent a standard psychiatric evaluation to diagnose TS.

The average age of the 15 patients was 28.3 years (SD = 7, min 19, max 40). Eight patients were transmen (FtMs—transman is assigned female at birth, but identifies as male) and seven were transwomen (MtFs—transwoman is assigned male at birth, but identifies as woman). MtFs and FtMs did not differ significantly in terms of age and duration of the sex-hormone therapy (all patients examined in the study commenced an endocrine treatment in 2–6-month duration).

### 2.2. Procedure

The Rorschach was administered to the patients in accordance with the procedures for the Comprehensive System and it is a standard diagnostic measure in TSP according to the Cabinet of Transgender States, Clinic for Psychiatry, Clinical Centre of Serbia, Belgrade [16].

### 2.3. Measures

#### 2.3.1. Rorschach Inkblot Method

Through the use of Exner's Comprehensive System in the analysis of Rorschach protocols, an overview of the seven clusters involving major domains of psychological functioning is obtained. Self-Perception cluster of variables provides information about how people view themselves, particularly with respect to their level of Self-Esteem, the extent of their Self-Awareness, and the nature of their Self-Image. The relevant Rorschach findings help to identify whether people feel satisfied and comfortable with themselves or are burdened by negative self-attitudes [18]. According to Weiner [18] this cluster has a composite structure and provides an insight into various aspects of the concept, such as the following.

*Maintaining Adequate Self-Esteem (Fr + rF, 3r + (2)/R).* Self-Esteem consists of the attitudes that people form toward their personal qualities and capabilities. The more favorably people compare themselves to others with respect to their qualities and capabilities, the higher their level of Self-Esteem is. Adequate Self-Esteem promotes Self-Acceptance, Self-Respect, and Self-Confidence based on realistic appraisal of one's capabilities, and it contributes to feeling of general satisfaction with themselves and their actions. Balanced attention to self and others and seeing oneself as being a worthy person are measured on the RIM by Reflections (*Fr* + *rF*) and the Egocentricity Index (3*r* + (2)/*R*).

*Promoting Positive Self-Regard (V, MOR).* Self-Regard is a composite of relatively positive and negative self-attitudes. The attitudes that constitute an individual's Self-Regard often fluctuate in response to changing circumstances. Used in concert with Reflections and the Egocentricity Index as measures of Self-Esteem, the Rorschach Vista (V) and Morbid (MOR) variables monitor aspects of Self-Regard.

*Enhancing Self-Awareness (FD).* Viewing oneself adaptively involves maintaining a moderate level of Self-Awareness. The FD variable provides a Rorschach Index of interest in and capacity for introspection.

*Forming a Stable Sense of Identity [H:Hd + (H) + (Hd)].* A stable Sense of Identity fosters good psychological adjustment by providing people a clear and consistent impression of the kind of individual they are, what they believe in, and where they are headed in their lives.

### 2.4. Data Analysis

Data are expressed as mean values for each of the variables taken into consideration in this study. The data were analyzed by using SPSS-18 statistical package (SPSS Inc., Chicago, Illinois, USA).

## 3. Results

The results of variables for the cluster examined which were obtained via Rorschach protocol are presented in Table 1; that is, these are the means and standard deviations for variables included in the Self-Perception cluster.

The analysis of the means and standard deviations for the variables of interest of the study shows no significant deviations from the average values for normal population compared to the Hypervigilance Index (HVI = 0) and Obsessive Style Index (OBS = 0) that are negative. The Egocentricity Index a measure of Self-Focusing which is about the average show no significant deviations, while 6 the values indicating negative Self-Regard (V = 0.866, MOR = 2.133) had a tendency to increase. The score related to the Enhancing Self-Awareness (FD = 1.73) indicates a tendency toward elevated Self-Inspection of transsexual persons. In addition, given that the average sum of *An* + *Xy* exceeds 1, it could be said that attention to an individual physical aspect is increased in TSP.

## 4. Discussion

The obtained average values of the seven variables included in the cluster of Self-Perception indicate potential ways in which people see themselves. None of the 15 survey respondents showed positive indexes, Hypervigilance Index (HVI) and Obsessive Style Index (OBS), which indicates that such persons have no prevailing tendency toward perfectionism, nor general vulnerability which is a result of basic mistrust towards the environment.

The values obtained in *Fr* + *rF* responses and Egocentricity Index 3*r* + (2)/*R* indicate Maintaining Adequate Self-Esteem. Replies indicating reflection were present only in one patient, and therefore the presence of evident narcissistic characteristics cannot be generally attributed to the examined sample of TSP. As for the Egocentricity Index that indicates the level of Self-Care, mean values were obtained as for normal adult population, ranging from 0.33 to 0.44; thus we cannot say that transsexual persons are self-centered to a greater extent than usual [18].

Values indicating the quality of Self-Regard (V = 0.866, MOR = 2.133) indicate that the Self-Inspection process comprised negative and painful emotional states, associated with chronic concerns of a negative Self-Image, which is often accompanied by negative emotions due to gender dysphoria. The average value of MOR responses suggests that negative features are included in the Self-Concept or that there is an experience of imperfection. This finding is consistent with the core psychological conflict between gender and sex underlying the transsexualism. Considering that the MOR responses represent materials of significant projective importance, there seems to be a trend showing that their Self-perception includes the subjective impression of a personal imperfection or inadequacy. Primarily negative Self-Regard contributes to adjustment problems associated with self-critical, self-denigrating, and self-loathing attitudes and also with depression and suicidality which is often observed as a trend in people with gender dysphoria.

Enhancing Self-Awareness (FD = 1.73) indicates a tendency toward elevated Self-Inspection and Self-Awareness, which suggests a presence of alert attention in relation to the intrapsychic experiences. People who are overly aware of themselves tend to be self-conscious to a fault. Constantly examining themselves and always alert to how they may look or sound to others, they have difficulty relaxing and just being themselves in a natural and unconcerned manner. Such finding, combined with the value of Sum Vista (V), indicates painful emotions related to introspection which is consistent with the experience of psychological discomfort conditioned by core issues in transsexuals.

Having in mind that the sum *An* + *Xy* exceeds 1, we can say that the attention to one's physical aspect is elevated. This finding is consistent with the diagnostic criteria and clinical experience in working with transsexual persons, who show a negative impression of their body and the physical structure because there is a strong sense of discomfort with one's body and desire to change the body to harmonize it with the subjective gender experience. Forming a stable Sense of Identity is connected with the capacity to identify oneself with real persons. Due to the fact that the number of *H* responses is greater than the *Hd* + (*H*)+(*Hd*), respondents who make up our sample meet these criteria and typically have adequate capacity to identify themselves with people who are a real part of their lives and with whom they have had opportunities to form such identifications.

## 5. Conclusion

Overall results of our study indicate that transsexual persons have Adequate Self-Esteem, while the Egocentricity Index is of average values as in normal adult population. They also indicate a trend toward elevated introspection, with increased attention to the physical aspects of the body; that is, in the process of Self-Inspection negative and painful emotional states are present. It means that the trend of increased number of MOR responses in relation to the studies on adolescents transsexual persons is probably the result of long-term suffering, having in mind that the people who are involved in our study had initiated the sex reassignment procedure during their mature adult age [17, 25]. Prolongation or entering the desired procedure relatively late results in the trend mentioned above, that is, the subjective experience of personal imperfection or inadequacy.

Analysis of the results obtained may indicate that delayed entering into the sex-reassignment procedure in transsexual persons may potentiate the negative image of themselves and cause exhaustion of adaptive resources and consequently development of comorbid disorders, particularly depression and suicidal behavior.

## 6. Limitations of the Study

The pilot study conducted has several limitations which need to be acknowledged. One of the limitations is the size of the sample. The total number of subjects was small for statistical purposes so only descriptive statistics was performed. Another limitation is the absence of a control group. According to the results obtained, in further research it is necessary to conduct a study that would include the assessment of Self-Perception in transsexual persons of different age groups, aimed at confirming the assumption that the prolongation of entering into the desired changes affects some aspects of Self-Perception.

## Figures and Tables

**Table 1:** Means and standard deviations for variables included in cluster of Self-Perception.

	Min	Max	M	SD
HVI	0	0	0	0
OBS	0	0	0	0
Index of Egocentricity	0.04	0.60	0.34	0.14
*Fr* + *rF*	0	2	0.13	0.51
Sum *V*	0	2	0.86	0.63
FD	0	5	1.73	1.33
*An* + *Xy*	0	7	1.80	1.93
MOR	1	5	2.13	1.12
*H*	1	4	2.53	1.06
*Hd* + (*H*)+(*Hd*)	0	5	2.06	1.57
